# EBG Based Microstrip Patch Antenna for Brain Tumor Detection via Scattering Parameters in Microwave Imaging System

**DOI:** 10.1155/2018/8241438

**Published:** 2018-02-12

**Authors:** Reefat Inum, Md. Masud Rana, Kamrun Nahar Shushama, Md. Anwarul Quader

**Affiliations:** Department of Electrical and Electronic Engineering, Rajshahi University of Engineering and Technology, Rajshahi, Bangladesh

## Abstract

A microwave brain imaging system model is envisaged to detect and visualize tumor inside the human brain. A compact and efficient microstrip patch antenna is used in the imaging technique to transmit equivalent signal and receive backscattering signal from the stratified human head model. Electromagnetic band gap (EBG) structure is incorporated on the antenna ground plane to enhance the performance. Rectangular and circular EBG structures are proposed to investigate the antenna performance. Incorporation of circular EBG on the antenna ground plane provides an improvement of 22.77% in return loss, 5.84% in impedance bandwidth, and 16.53% in antenna gain with respect to the patch antenna with rectangular EBG. The simulation results obtained from CST are compared to those obtained from HFSS to validate the design. Specific absorption rate (SAR) of the modeled head tissue for the proposed antenna is determined. Different SAR values are compared with the established standard SAR limit to provide a safety regulation of the imaging system. A monostatic radar-based confocal microwave imaging algorithm is applied to generate the image of tumor inside a six-layer human head phantom model. *S*-parameter signals obtained from circular EBG loaded patch antenna in different scanning modes are utilized in the imaging algorithm to effectively produce a high-resolution image which reliably indicates the presence of tumor inside human brain.

## 1. Introduction

Brain cancer is one of the serious public health problems worldwide because it affects the most vital organ of human body. For example, in the USA, 23,800 patients and 16,700 deaths are estimated due to brain cancer in 2017 [1]. Such a high death rate is caused by the invasive properties of tumors which turn brain cancer into a serious disease. But it is encouraging that the cure rate can be increased by reliably diagnosing it in the early stages because treatment at early stage is more efficient and effective compared with treatment done at the late stage of cancer. The common imaging modalities utilized to detect cancer are magnetic resonance imaging (MRI) scanning, X-ray screening, computed tomography (CT) scans, positron emission tomography (PET), and ultrasound imaging [2]. The possibility of microwave imaging technology for brain cancer detection is increased recently as it offers a safe, rapid, low-cost, noninvasive, and highly accurate system solution which involves nonionizing radiation [3].

Microwave imaging is an active wave-based noninvasive imaging method. Nonionizing electromagnetic waves from microwave signals are able to penetrate human tissues without creating health hazards [4]. The contrast in the electrical properties between healthy and malignant tissues is the principal of operation of microwave imaging systems. Four large groups, namely, optimization-based microwave imaging, microwave tomography, confocal radar-based imaging, and microwave holography can be considered as the branches of currently active microwave systems for tissue imaging [5]. Radar-based techniques are preferable since they only focus on detecting the tumor rather than the entire range of electrical properties [6]. Therefore, much easier signal processing such as less sophisticated delay and sum confocal microwave imaging algorithm is involved in radar-based microwave imaging.

In any brain imaging system, the antennas form an important element for the quality of the final image and the performance of imaging. The appropriate antenna for one such system should cover a number of requirements including ease of integration, compactness, simple geometric structure, small dimensions, enhanced bandwidth, gain, and directionality [7–11]. These requirements can be fulfilled by a microstrip patch antenna because it possesses a number of advantages like low profile, light weight, low volume, planar configuration, low fabrication cost, and so forth [12]. However, conventional patch antennas need some modifications for reliable pulse transmission and collection of backscattered signals in microwave imaging systems. Amalgamation of electromagnetic band gap (EBG) structure on the antenna ground plane is found in the literature to be an effective way to enhance the performance of conventional microstrip patch antenna [13]. EBG structures are any artificial periodic objects designed to prevent/assist electromagnetic wave propagation in a given band of frequency. Due to their unique band gap features, EBG structures can be categorized as a special type of metamaterial. This unique property has been applied to design antenna systems with better gain and efficiency, reduced mutual coupling, and lower side-lobes and back-lobe levels by suppressing surface wave modes [14, 15].

Assessment of specific absorption rate (SAR) induced inside human head is another important feature of any microwave brain imaging system. EM waves radiated from the transmitting antenna directly travel through the patients head and significant portion of the radiated power carrying by EM waves is absorbed by head tissues. These energy absorptions are not distributive in nature and may cause localized RF energy deposition in the form of nodes and damage DNA of living tissues which may trigger cell suicide or unregulated cell division resulting in the formation of a cancerous tumor [16]. Hence accurate SAR analysis which greatly depends on the exact head phantom model is a must one in imaging system to ensure the safety of patient under test. As the precise modeling of multilayered human head consisting of different complex tissues is cumbersome, various head phantom models have been investigated to mimic the practical scenario [17–19].

The goal of this study is twofold: (1) design verification of an EBG based microstrip patch antenna by comparing the results obtained from CST and HFSS software and (2) detection of tumor inside human brain by radar-based microwave brain imaging system. In [9], the simulation results obtained from CST showed that the patch antenna with circular EBG structure provides best performance and consequently it is applied in the brain tumor detection application. The normal six-layered spherical human head phantom modeled in [9] is made, a cancerous phantom by inserting a tumor model inside head. The dimensions and electrical properties of head and tumor models are chosen feasibly that reflect the actual ones. To get the imaging results, proposed antenna is simulated with head phantom model by CST MWS software. The *S*-parameter results obtained from different scanning modes involving head phantom with and without tumor model are applied in the microwave imaging algorithm to reconstruct tumor image. The SAR values for three different measuring techniques are compared with each other and with the established standard SAR limit so that safety of microwave brain tumor detection system is ensured. Thus, EBG based microstrip patch antenna is applied in brain tumor detection for the first time with all the necessary aspects of microwave brain imaging system being clearly analyzed.

## 2. Radar-Based Microwave Brain Imaging System Model

**Figure 1:**
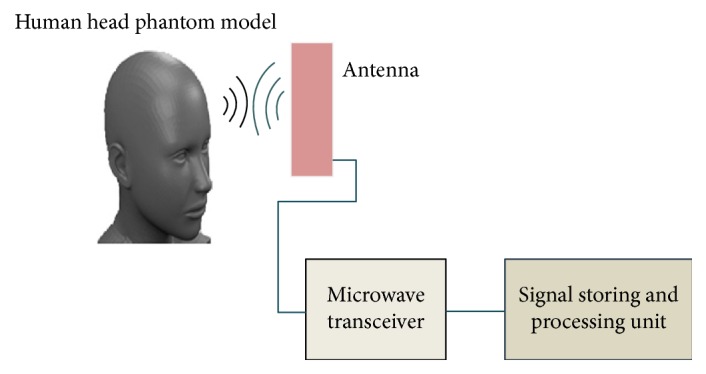
Schematic diagram of monostatic radar-based microwave brain imaging system [9].

The aim of radar-based imaging method is to use backscattering signals for the creation of images which can detect the presence of an object embedded in a dielectric medium. The received signal is stored and a suitable signal processing algorithm makes the image possible.  Figure 1 depicts the concept of our considered brain imaging system [9]. In this system model, we specifically focus on the effective antenna modeling, human head phantom modeling, detection of tumor inside human brain by radar-based microwave image reconstruction algorithm, and determination of SAR values inside six-layer human head model.

The head-imaging architecture shown in Figure 1 primarily consists of a compact antenna to transmit and receive wideband signals, a microwave transceiver for signal generation, and data acquisition and a personal computer for signal processing and image formation. A head-imaging platform is designed virtually in CST MWS software to evaluate the system's performance on detecting tumor using a realistic head phantom. The head phantom is scanned by changing the antenna position in different manner and the reflected signals are collected and converted from frequency domain to time domain. The resulting signals are then processed and used in confocal microwave image reconstruction algorithm to visualize the presence of tumor inside human head.

### 2.1. Antenna and EBG Structure Design

The development of microstrip patch antenna with EBG structure and relevant theory is discussed in this section. Initially, a rectangular microstrip patch antenna is designed without any EBG structure and taking 7.3 GHz as the resonant frequency. The reason behind the choice of such working frequency is twofold. Firstly, higher frequency helps to improve the spatial resolution of the reconstructed tumor image and, secondly, relatively larger resonant frequency can ensure a compact antenna with small dimension, which is important for any brain imaging system. The antenna is designed on Rogers R03003 substrate with relative permittivity of 3 and with the thickness and dimension of 0.75 mm and 31.68 mm × 31.02 mm, respectively. Due to its flexibility for high frequency application compared to FR4, Rogers R03003 is chosen as the substrate material [9]. However, the performance of an antenna is greatly affected by substrate thickness and it is known that a thicker substrate can enhance the antenna efficiency and bandwidth but in exchange a surface wave is initiated which reduces the amount of radiated power [20].

Figure 3 shows the geometry of the patch antenna without EBG structure, where top view of the antenna is illustrated with all necessary dimension parameters. The detail design procedure with all the essential equations is available in [20]. Microstrip line feeding technique is used to feed the antenna and 50 Ω is taken as the characteristic impedance of the transmission line. Next, two different types of EBG structures are proposed to improve the antenna performance. Incorporation of EBG structures on the antenna ground plane results in a reduction of patch length, substrate length, inset gap, and an increase in the substrate height.  Table 1 shows all the dimension parameters to design the patch antenna without EBG and optimized antenna with circular EBG structure. The geometry of the patch antenna with EBG structure is shown in Figure 4(a). The dimensions of EBG structures are determined from the wavelength of the microwave signal radiated by the proposed antenna. Each side of the rectangular EBG and diameter of the circular EBG are calculated as 0.1*λ*, as can be seen in Figures 4(b) and 4(c), respectively. The distance between two adjacent EBGs in either case is determined as 0.02*λ*, which is evident from Figures 4(b) and 4(c).

In designing the proposed EBGs, Nicholson-Ross-Weir (NRW) technique [21] is used as a conversion approach to obtain both the permeability and permittivity from the *S*-parameter. The relative material parameters can be calculated as follows:

(1) n2=εrμr=−cωdln⁡eγd2+ωcω2,μr=1+Γ1−Γn2−ωc/ω21−ωc/ω2,

where *n* is the refractive index, *ε*_*r*_ is the relative permittivity, *μ*_*r*_ is the relative permeability, *d* is the maximum length of metamaterial unit cell, $c=1/\sqrt{\varepsilon_{0}\mu_{0}}$ is the speed of light in free space, *ω* is the angular frequency, and *ω*_*c*_ is the angular cutoff frequency of the incident wave on the metamaterial. The inverse to the quantity *e*^*γd*^ is known as the phase factor for electromagnetic (EM) wave propagating through the metamaterial and is given by

(2) $$\begin{array}{c} e^{-\gamma d}=\frac{S_{11}+S_{21}-\Gamma }{1-\left(S_{11}+S_{21}\right)\Gamma }, \end{array}$$

where $\gamma =j\omega \sqrt{\varepsilon_{0}\mu_{0}}\sqrt{n^{2}-{\left(\omega_{c}/\omega \right)}^{2}}$ is the propagation constant and Γ is given by

(3) Γ=X±X2−1,

(4) X=S112−S212+12S11,

where *S*_11_ and *S*_21_ are the reflection and transmission coefficients which are collected by simulating the metamaterial unit cell. These reflection and transmission coefficients are utilized in the above equations to obtain the permittivity and permeability curves as shown in Figures 5(a) and 5(b). These figures show that the designed EBG structures work as metamaterial as they provide negative permittivity and permeability in the desired frequency of 7.3 GHz.  Figure 5(a) shows the extracted material parameters (*ε* and *μ*) for the circular EBG structure, which indicate that both the permittivity and permeability are negative in the frequency range of 3 GHz to 8 GHz. For the rectangular EBG structure, the extracted parameters (*ε* and *μ*) are negative in the frequency range of 6.6 GHz to 7.8 GHz, as evident from Figure 5(b). In both the cases, the antenna resonance frequency (7.3 GHz) falls in the range of frequency for which the extracted permittivity and permeability are negative. Therefore, the proposed rectangular and circular EBG structures are suitable for incorporation on the ground plane of the designed patch antenna and are expected to improve the antenna performance by sufficiently reducing the unwanted surface wave.

### 2.2. Modeling of Cancerous Human Head Phantom

The frequency dependent dielectric properties (permittivity and conductivity) of the designed head phantom must imitate the properties of real human head so that the interaction between EM waves and head tissues can be correctly analyzed. For the radar-based microwave brain imaging, the phantom design can be simplified in an approximation as a multilayer model. In [9], a six-layered spherical head phantom that mimics the realistic human head is modeled for the measurement of specific absorption rate (SAR). In this paper, the healthy head phantom in [9] is made cancerous by inserting a tumor inside the brain, as shown by a schematic representation in Figure 6.

A human brain model with tumor inside it, as shown in Figure 6, is used in the simulation. The tumor is placed in between CSF and dura close to the bone. The radiated signals from the antenna start penetrating the outer tissue layer and gradually move toward the inner layer. However, attenuation of RF energy occurs very quickly due to backscatter from each tissue layer. Therefore, the tumor is placed close to the bone so that it can be easily detected from the simulation results. In contrast, detection of heavily buried tumor inside multilayer human brain becomes difficult as the reflected signal from tumor in this case could be too weak. The dimension and dielectric properties of all head tissues and tumor model are given in Table 2 [17, 18].

Considering the above realistic dielectric tissue properties in the frequency band of interest, tumor and a six-layered heterogeneous head phantom are modeled to simplify the simulation study. In monostatic radar-based microwave imaging technique, a single antenna configuration is used to scan the brain phantom with backscattering signal recorded at each antenna position.  Figure 7 illustrates the simulation setup for the considered brain imaging method where the proposed microstrip patch antenna is located at 10 mm distance from the phantom surface for suitable penetration of microwave signal inside the tissue layers.

## 3. Results and Discussions

The performance of the designed antennas is initially evaluated using Finite Integration Technique (FIT) based CST Microwave Studio (MWS) software and later verified by Finite Element Method (FEM) based HFSS software. Reflection coefficient, gain, and bandwidth are considered as the antenna performance parameters. After analyzing the antenna performance from microwave imaging perspectives, backscattering signals are collected and processed by confocal microwave imaging algorithm to reconstruct tumor image. Finally biocompatibility analysis is conducted by assessment of SAR induced inside the cancerous head to determine whether the considered imaging system is prone to health hazard or not.

### 3.1. Performance Analysis of the Designed Antennas

For better impedance matching, the reflection coefficient of an antenna must be at least −10 dB. Specifically, for the antennas to be applied in a brain imaging system, the reflection coefficient should be more negative to ensure minimal reflection of the input power from antenna to the source. The resolution of the reconstructed image in microwave imaging depends on the scattered signal power which is proportional to the radiated power [9]. The amount of total radiated power from the scanning antenna depends on the input power which will be maximum if the reflection coefficient is enhanced. Therefore, the quality of the reconstructed image can be somewhat improved by enhancing the reflection coefficient of the scanning antenna. The performances of the designed antennas in terms of reflection coefficients are depicted in Figure 8.

It is observed from Figures 8(a), 8(b), and 8(c) that the reflection coefficients obtained from CST closely agree with that obtained from HFSS, for the patch antenna without EBG, with rectangular EBG, and with circular EBG, respectively.  Figure 8(d) compares the reflection coefficients for three antennas, which indicate that the normal patch antenna and patch antenna with rectangular EBG and with circular EBG provide reflection coefficient of −18.40 dB, −40.15 dB, and −49.29 dB, respectively. Therefore, the incorporation of circular EBG structure on the antenna ground plane offers the best result in terms of reflection coefficient. The information on impedance bandwidth for the three antennas can also be obtained from Figure 8, which show that the patch antennas without EBG, with rectangular EBG, and with circular EBG provide impedance bandwidth of 156.20 MHz, 275.50 MHz, and 291.60 MHZ, respectively. Hence in this case also, antenna with circular EBG gives the best performance among other designs.

Besides impedance bandwidth, the performance of the designed antennas is also investigated in terms of gain bandwidth.  Figure 9 shows the gain of the designed antennas as a function of frequency. It is observed from Figure 9 that the patch antenna with circular EBG provides a flat gain of approximately 6.7 dBi from 6 to 8 GHz, while the normal patch antennas without EBG and antenna with rectangular EBG provide approximately a flat gain of 6.06 dBi (6.4 to 7.4 GHz) and 5.89 dBi (6.3 to 7.4 GHz), respectively. It can be noted that the working frequency (7.3 GHz) falls in the range of frequencies over which all the three antennas provide a flat gain bandwidth. Also, the antenna with circular EBG structure offers the largest flat gain bandwidth in comparison to the other two antennas. Therefore, signal transmission and reception with reduced distortion and dispersion can be attained by the circular EBG based antenna which makes it a potential candidate for the envisaged brain imaging application.

The *E*-plane (*φ* = 90°) radiation patterns of the designed antennas in terms of gain are shown in Figure 10(a). It is evident from this figure that there is a close agreement between the *E*-plane patterns obtained from CST and HFSS, which validate the design. Figure 10(a) displays that the normal patch antenna without EBG and antenna with rectangular EBG and with circular EBG provide a gain of 6.17 dBi, 5.91 dBi, and 6.77 dBi, respectively, at 7.3 GHz. The main lobe of the *E*-plane pattern in terms of enhanced gain for antenna with circular EBG is directed at *θ* = 20°. Radiation characteristics like 3 dB beamwidth and side-lobe level for circular EBG based patch antenna are 92.6 degree and −8.9 dB. The enhancement in the radiation characteristics for antenna with circular EBG structure is particularly important from imaging perspectives, because it ensures reduced distortion and dispersion in signal transmission and reception and increased resolution capabilities of the scanning antenna.

Figure 10(b) shows the *H*-plane (*φ* = 0°) radiation patterns in terms of gain at 7.3 GHz for the three antennas. It is obvious from this figure that the patterns obtained from CST and HFSS closely agree with each other for all the three designs, which further indicate a validation of the design. A comparison of the *H*-plane patterns between three antennas is also provided in Figure 10(b) which reveals that the antenna with circular EBG performs the best (6.36 dBi) in comparison to the antenna without EBG (5.92 dBi) and with rectangular EBG (5.17 dBi). It is also observed from Figure 10(b) that the main lobe direction for all the three antennas is at *θ* = 0°, which is parallel to the *z*-axis and thereby facilitates positioning the antenna in the scanning mechanism. However, the improved gain offered for the circular EBG based patch antenna in both the planes is a consequence of the unique structural feature of circular shaped EBG which strongly reduces the surface waves produced on the antenna substrate.

Finally, a comparison between the performance parameters of the proposed antenna and similar other existing antennas is given in Table 3. Here, microstrip patch antenna with circular EBG structure is proposed for the considered brain imaging application, due to its improved performances as evident from the above discussion. Similar other antennas mean those designed incorporating any metamaterial structure [14, 22, 23] or antennas designed for brain imaging application [10, 11]. It is obvious from Table 3 that the proposed microstrip patch antenna with circular EBG structure possesses a compact size in comparison to other antennas, which is extremely beneficial for microwave brain imaging system. It is also evident from the comparison table that the antenna proposed in this paper outperforms similar other antennas with respect to gain and reflection coefficient. In the case of bandwidth, the proposed antenna offers somewhat little impedance bandwidth of 0.29 GHz, the reason of which may be the use of a very thin (35 *μ*m) conductor. However, the antenna designed in [11] has been utilized in head-imaging application, with a measured impedance bandwidth of 0.26 GHz. Therefore, circular EBG based patch antenna proposed in this paper can be reliably applied in microwave brain imaging, with an impedance bandwidth of 0.29 GHz.

### 3.2. Brain Tumor Detection

**Figure 2:**
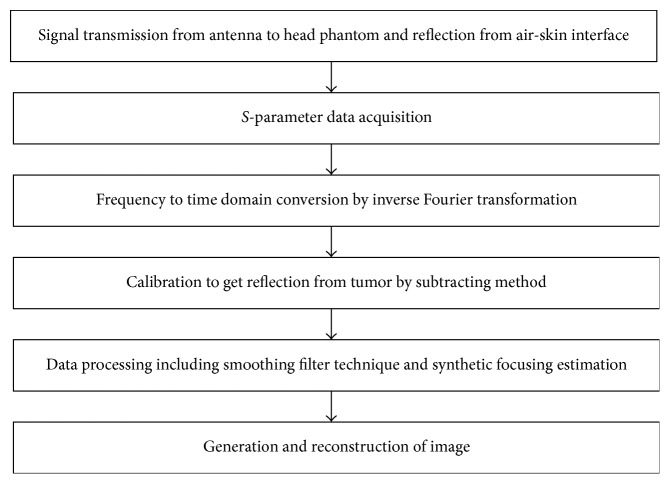
Confocal microwave image reconstruction algorithm for the considered brain imaging system model.

**Figure 3:**
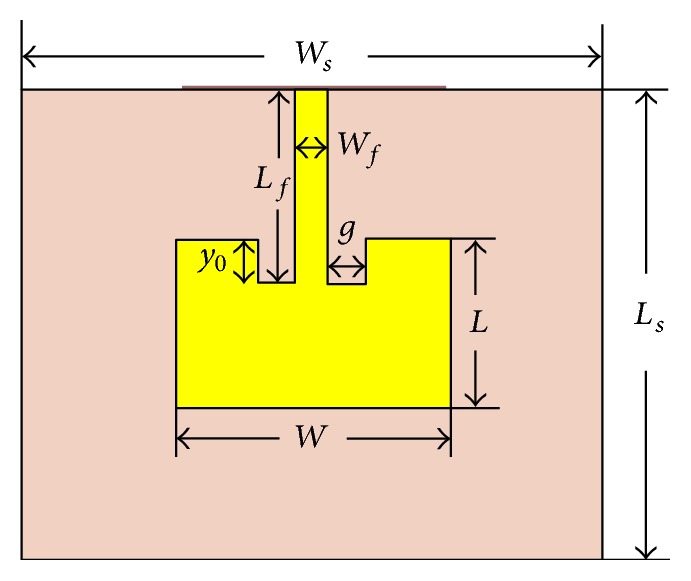
Schematic diagram of patch antenna without EBG structure.

**Figure 4:**
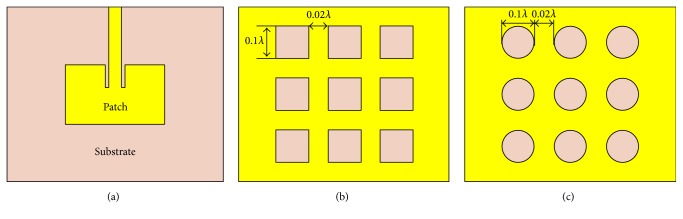
Schematic diagram of (a) patch antenna with EBG; (b) geometry of rectangular EBG; (c) geometry of circular EBG.

**Figure 5:**
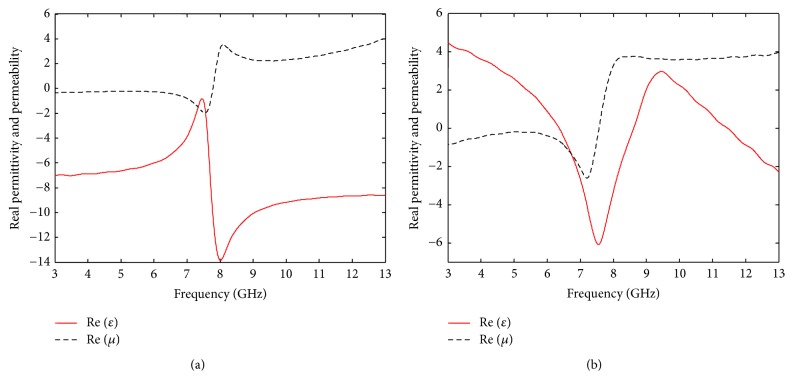
Real permittivity and permeability of (a) circular and (b) rectangular EBG structures.

**Figure 6:**
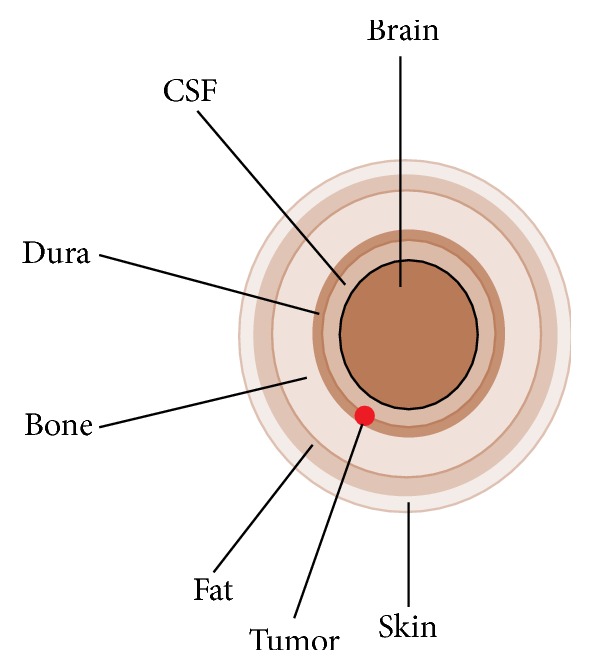
Schematic representation of the designed spherical cancerous head phantom.

**Figure 7:**
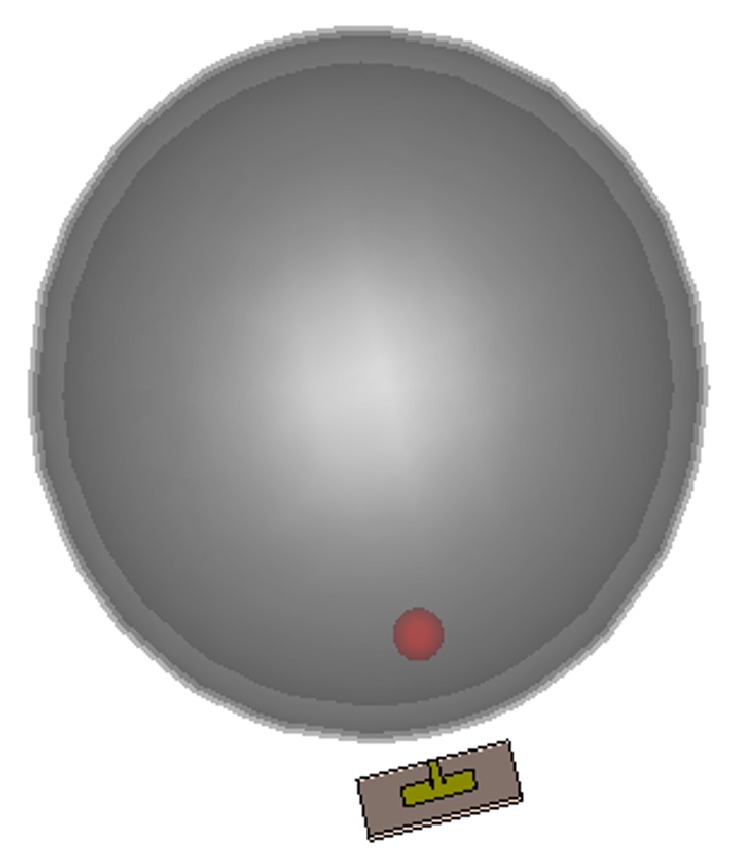
Simulation setup for the considered brain imaging method.

**Figure 8:**
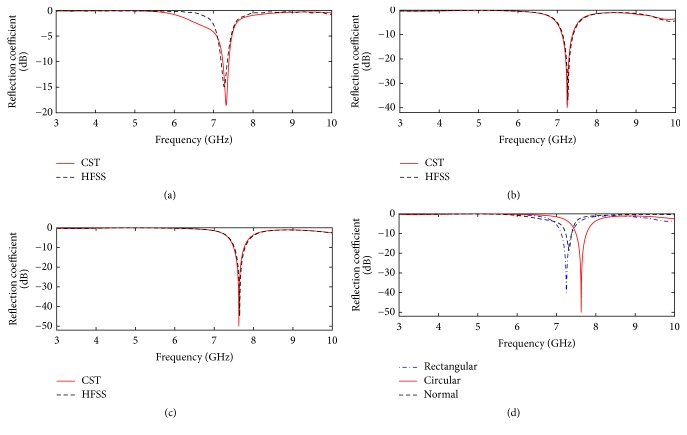
Reflection coefficients of the patch antenna (a) without EBG, (b) with rectangular EBG, (c) and with circular EBG and (d) comparison between three antennas.

**Figure 9:**
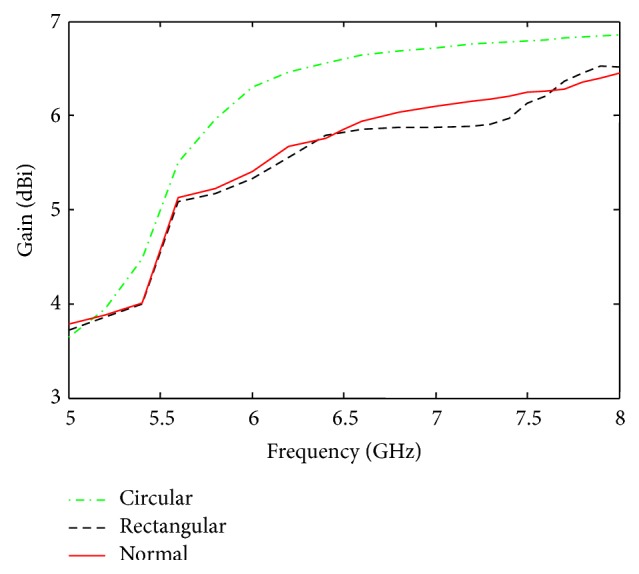
Gain of the designed antennas as a function of frequency.

**Figure 10:**
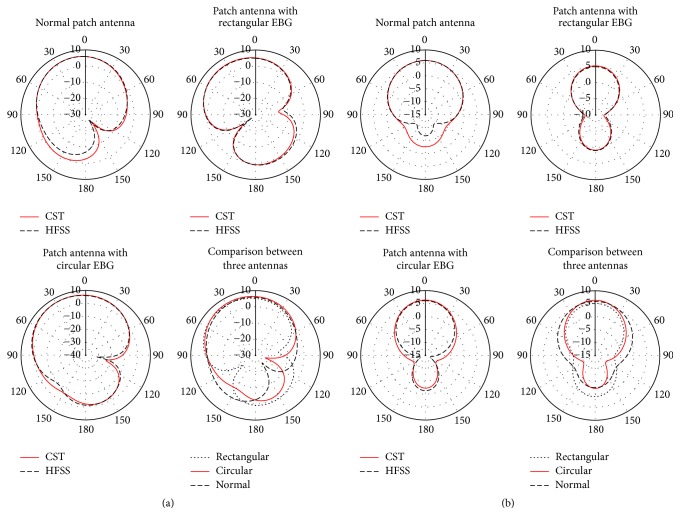
Radiation patterns of the designed antennas at 7.3 GHz in (a) *E*-plane and (b) *H*-plane.

**Figure 11:**
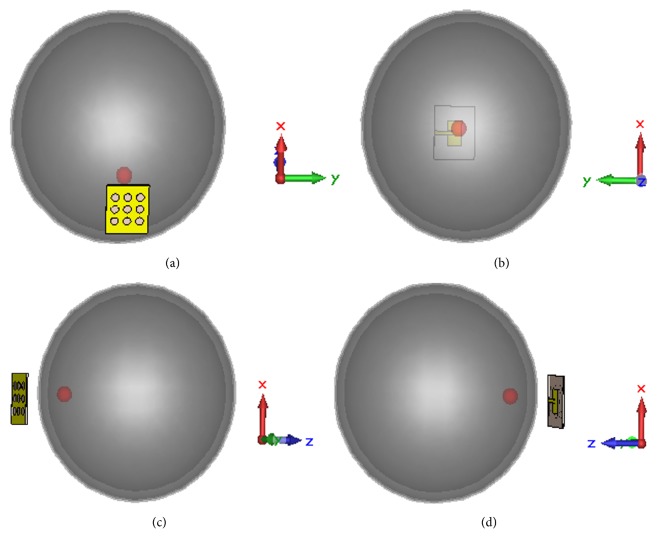
Position of antenna for scanning the phantom in (a) *x*-*y* plane, (b) reverse *x*-*y* plane, (c) *x*-*z* plane, and (d) reverse *x*-*z* plane.

**Figure 12:**
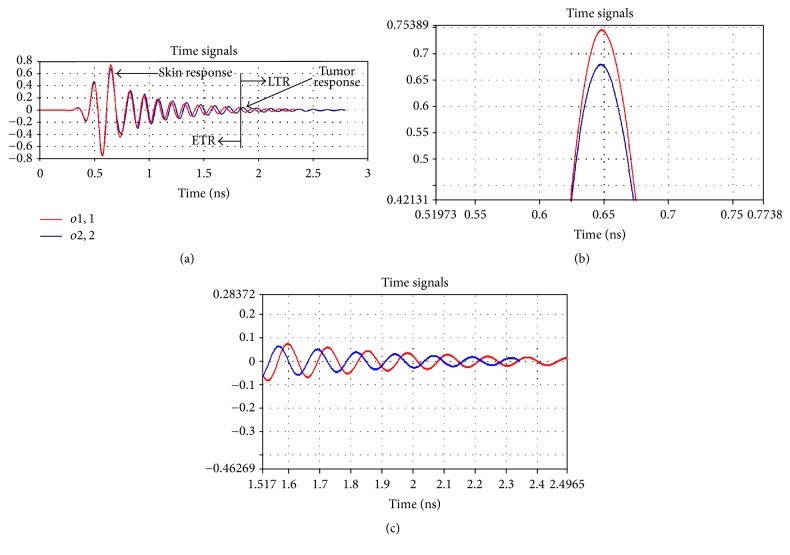
(a) Reflected transient signal by simulating antenna using head phantom with and without tumor, (b) enlarged skin response, and (c) enlarged tumor response.

A sequence of task is done as depicted in the algorithm in Figure 2 to detect the tumor inside head phantom using a monostatic radar-based confocal microwave image reconstruction system. Monostatic radar-based technique adopted in this work is based on the concept where a single antenna scans the head phantom by mechanically rotating around it. However, in CST simulation environment, mechanical rotation is accomplished by moving the antenna to all the possible locations around the phantom and simulating the system for each antenna location.  Figure 11 illustrates the antenna positions around the head phantom in different scanning mode. Figures 11(a) and 11(c) show the scanning in *x*-*y* and *x*-*z* plane. To get a clearer visualization of the 3D system in 2D plane, Figures 11(b) and 11(d) provide scanning performance in reverse *x*-*y* and reverse *x*-*z* plane. In all these scanning modes, multiple reflections from different head tissue layers and tumor response, and noise are contained in the collected scattering signals. As the microwave signal reaches tumor after penetrating some complex tissue layers, sufficient signal attenuation occurs which make tumor response quite weak resulting in an easy drowning out in the noise. Therefore, signal processing is a must one to remove all the unwanted signals which results in a high-resolution image with low noise level for reliable diagnosis. A reference transient scattering signal using a head phantom without tumor being presence is needed to remove all unwanted parts of signal. By subtracting the reference signal from originally received signal, tumor response can be extracted. To obtain the reference and original signals, proposed antenna is simulated using head phantom with and without tumor. Reflection coefficient for original and reference signals are shown in Figure 12(a). *o*1,1 and *o*2,2 represent the reflected energy from head phantom with and without tumor, respectively. ETR and LTR stand for early time response and late time response, respectively. When a cancerous human head is illuminated by an incident EM wave, skin and other head tissue layer contained in the scattering wave involve the optical region, which means that these reflections contribute to the ETR. The tumor reflection is involved with the resonance region and contributes to the LTR. Hence, skin and tumor responses can be extracted by investigating the turn-on time of LTR which is defined as twice the time taken by incident EM wave to pass through the surface of the object [24].  Figure 12(b) shows that the energy reflected from skin layer of head phantom with tumor is greater than that without tumor. The strong reflection from the high permittivity tumor tissue is the reason behind this result. Enlarged portion of tumor response is illustrated in Figure 12(c) which shows that reflected transient signal for head phantom without tumor becomes steady state quicker than that with tumor.

**Figure 13:**
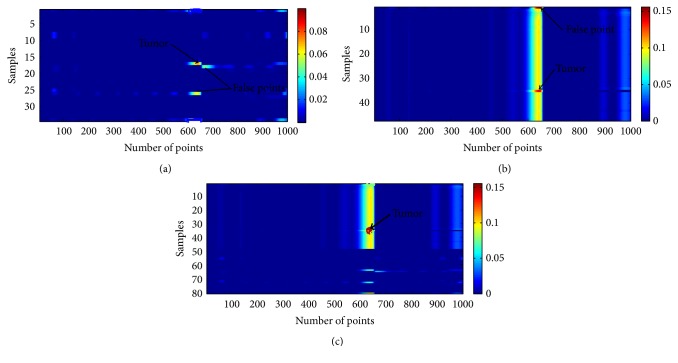
Reconstructed microwave image of tumor by scanning head phantom in (a) *x*-*y* plane, (b) *x*-*z* plane, and (c) *x*-*y* and *x*-*z* plane.

From the above discussion it is clear that the reflected energy depends on the presence of tumor which in turn depends on antenna position. This basic principle is used to generate microwave images by moving the antenna to different locations surrounding the spherical human head phantom, with scattering signals recorded at each stop location. The images are created by using the intensities of the scattering signals at each scan position. The resulting microwave image of tumor inside multilayer human head phantom is shown in Figure 13. Presence of tumor with two false points can be observed in Figure 13(a) where scanning is performed in *x*-*y* plane. Due to reflections from different tissue layers in a cylindrical scanning mode in *x*-*y* plane, presence of two false points and a nonspherical shaped tumor is evident from Figure 13(a). When the same rotational scanning is done in *x*-*z* plane, resolution of image is increased and the number of false points is decreased, as shown in Figure 13(b). This improvement is accomplished because the pulse from antenna in this case is transmitted to a more exact *z* direction. However, the reconstructed image still contains some noise in the form of false point due to the inability of radiation pattern of the antenna to cover the whole round phantom. To overcome these difficulties, a combined rotational scanning is carried out where the data from rotational scanning in *x*-*y* and *x*-*z* plane are combined together. The reconstructed image for combined scanning mode is shown in Figure 13(c) where the resolution and shape of tumor are fairly good with minimal possibility of false diagnosis. Therefore microwave imaging via combined rotational scanning of a multilayer human head phantom can reliably detect the presence of tumor inside the human head.

### 3.3. Evaluation of Specific Absorption Rate (SAR)

**Figure 14:**
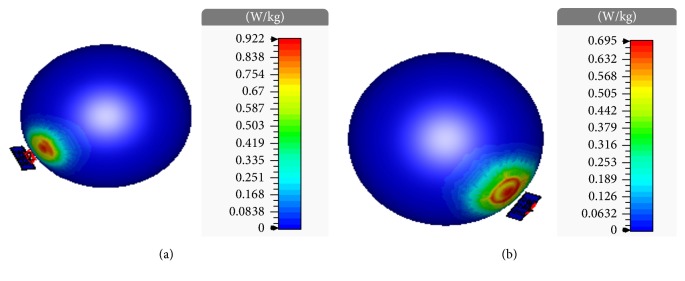
(a) 1 g average and (b) 10 g average SAR induced inside the human head tissue.

As a brief exposure to radiation may cause severe health hazard, the safety regulation of the envisaged brain imaging system must be maintained by proper assessment of SAR. The deposition of electromagnetic energy over time into human body tissue is defined as a measure of SAR [25]. The mathematical expression of SAR is as follows [25]:

(5) $$\begin{array}{c} SAR=\frac{\sigma }{\rho }{\left|\;E\;\right|}^{2}=\frac{J^{2}}{\rho \sigma }, \end{array}$$

where *E* is the rms value of the electric field strength in the tissue, *J* is the current density, *σ* is the conductivity of the head tissue, and *ρ* is the density of head tissues. The unit of SAR is usually represented as watt per kilogram. Whenever human body is exposed to EM radiation, the induced SAR inside the body tissue can be assessed using different averaging techniques. The factors affecting SAR calculation are the operating frequency, type of antenna, and distance between the scanning antenna and exposed surface of human body. The international standard limit to SAR varies according to national reporting, testing requirement, and the network band. The 1 g average and 10 g average SAR limt are 1.6 W/kg and 2 W/kg, set by US and EU standard, respectively [26]. The distribution of SAR induced inside the six-layered head tissues is shown in Figure 14, which is a 3D plot obtained by numerical method based EM simulation. To calculate the mass averaged SAR, the power loss density is integrated over a cube with a defined mass of 1 g or 10 g and then the integral power loss is divided by the cube's mass [9]. The 1 g average and 10 g average SAR distributions obtained by such numerical calculations are displayed in Figures 14(a) and 14(b), respectively. The 3D maximum SAR values are 0.922 W/kg and 0.695 W/kg, as shown in Figures 14(a) and 14(b), respectively. It is clear from this figure that 1 g and 10 g average SAR values are well below the maximum standard SAR limit, which ensures the safety of our considered microwave brain imaging system. It can also be noted from Figure 13 that the SAR values are higher in the area of phantom surface near the antenna and quickly decreases to zero in the distant areas of head phantom surface. Therefore, no health risk regarding SAR is likely to occur for the envisaged monostatic radar-based microwave brain imaging system which makes it a potential and reliable tool to detect human brain tumor.

## 4. Conclusion

A brain imaging system working in microwave frequency range for human brain tumor detection is envisaged. An efficient microstrip patch antenna is devised whose performance is improved by slotting circular EBG structure on the antenna ground plane. The unique band gap feature of circular EBG configuration boosts antenna performance parameters, namely, reflection coefficient, gain, and bandwidth by considerable suppression of surface wave introduced on antenna substrate. The validation of the design is carried out by simulating the antennas with another software HFSS and comparing the results. The optimized and enhanced patch antenna based on circular EBG is applied in the considered imaging application. The antenna is used to scan a spherical six-layer human head phantom model. The *S*-parameter data obtained from a combined rotational scanning mode performed in *x*-*y* and *x*-*z* plane is utilized in a confocal microwave imaging algorithm to detect tumor inside the human brain. Finally, assessment of specific absorption rate (SAR) induced inside the human head phantom is carefully done. The overall results obtained confirm that the studied brain imaging system can successfully diagnose brain tumor at an early stage while maintaining safety regulation of the patient under test.

## Figures and Tables

**Table 1:** Rectangular microstrip patch antenna specifications.

Design parameters	Dimensions without EBG (mm)	Optimized dimensions with circular EBG (mm)
Patch width, *W*	14.53	14.53
Patch length, *L*	11.53	8.95
Inset feed, *y*_0_	3.125	3.41
Inset gap, *g*	1.901	0.38
Feed length, *L*_*f*_	13.202	11.895
Feed width, *W*_*f*_	1.901	1.9010
Substrate width, *W*_*S*_	31.02	31.0170
Substrate length, *L*_*S*_	31.68	25.92
Substrate thickness, *h*	0.75	1.03

**Table 2:** Dielectric properties of the spherical cancerous head phantom with approximate dimension.

Tissue	Radius (mm)	Permittivity (*ϵ*_*r*_)	Conductivity, *σ* (S/m)
Brain	81	43.22	1.29
CSF	83	70.1	2.3
Dura	83.5	46	0.9
Bone	87.6	5.6	0.03
Fat	89	5.54	0.04
Skin	90	45	0.73
Tumor	5	55	7

**Table 3:** Performance comparison of the proposed antenna with other existing antennas.

Parameter	Proposed antenna	[10]	[11]	[14]	[22]	[23]
Patch dimension (mm^2^)	14.53 × 8.95	50 × 37.5	25 × 25	28 × 28	14 × 14	15.25 × 9.07
Reflection coefficient (dB)	−49.289	NR	−20	−22	−18	−17.50
Gain (dBi)	6.77	2.6	6.6	2.2	2.5	5.01
Bandwidth (GHz)	0.29	2.16	0.26	0.03	0.19	2.89

NR: not reported.
